# OsWAK112, A Wall-Associated Kinase, Negatively Regulates Salt Stress Responses by Inhibiting Ethylene Production

**DOI:** 10.3389/fpls.2021.751965

**Published:** 2021-10-05

**Authors:** Wei Lin, Yuehua Wang, Xinye Liu, Jian-Xiu Shang, Liqun Zhao

**Affiliations:** Hebei Key Laboratory of Molecular and Cellular Biology, Key Laboratory of Molecular and Cellular Biology of the Ministry of Education, Hebei Collaboration Innovation Center for Cell Signaling, College of Life Sciences, Hebei Normal University, Shijiazhuang, China

**Keywords:** *Oryza sativa*, salt, wall-associated kinase, ethylene, S-adenosyl-L-methionine synthetases

## Abstract

The wall-associated kinase (WAK) multigene family plays critical roles in various cellular processes and stress responses in plants, however, whether WAKs are involved in salt tolerance is obscure. Herein, we report the functional characterization of a rice WAK, *WAK112*, whose expression is suppressed by salt. Overexpression of *OsWAK112* in rice and heterologous expression of *OsWAK112* in *Arabidopsis* significantly decreased plant survival under conditions of salt stress, while knocking down the *OsWAK112* in rice increased plant survival under salt stress. OsWAK112 is universally expressed in plant and associated with cell wall. Meanwhile, *in vitro* kinase assays and salt tolerance analyses showed that OsWAK112 possesses kinase activity and that it plays a negative role in the response of plants to salt stress. In addition, OsWAK112 interacts with S-adenosyl-L-methionine synthetase (SAMS) 1/2/3, which catalyzes SAM synthesis from ATP and L-methionine, and promotes OsSAMS1 degradation under salt stress. Furthermore, in *OsWAK112*-overexpressing plants, there is a decreased SAMS content and a decreased ethylene content under salt stress. These results indicate that OsWAK112 negatively regulates plant salt responses by inhibiting ethylene production, possibly *via* direct binding with OsSAMS1/2/3.

## Introduction

As sessile organisms, plants are more susceptible than animals to abiotic stresses, such as salinity, drought, and temperature extremes (Munns and Tester, 2008). Salt stress, an important abiotic stress, can affect all aspects of plant development (Hanin et al., 2016). The main manifestations of salt stress are reduced seed germination, decreased chlorophyll synthesis, premature senescence, and death after long-term exposure to excessive salt (Zhu, 2002). To withstand salt stress, plants have evolved a network of physiological, biochemical, and molecular mechanisms (Hanin et al., 2016). These mechanisms help minimize the harmful effects of ionic stress by sequestering Na^+^ in the vacuoles or outside of the cell *via* the classical salt overly sensitive pathway (Deinlein et al., 2014). Osmotic regulators are synthesized that counteract the osmotic stress created by excess salt (Yang and Guo, 2018). Plants may also increase the gene expression and protein activity of reactive oxygen species (ROS)-scavenging enzymes to alleviate oxidative damage (Sreenivasulu et al., 2004; Zhou et al., 2018). In addition, plants can induce the expression of salt stress-related genes to enhance their ability to adapt to high-salt environments (Huang et al., 2012). Still, a comprehensive investigation of the mechanisms that underlie plant cell responses to salt stress is required to understand how plants grow and survive under high-salt conditions.

The phytohormones play a crucial role in plant growth and development, and a sophisticated and efficient role under different environmental conditions. Among the nine well-characterized plant hormones, ethylene, gibberellin, jasmonic acid, and cytokinins are able to be regulated by salt stress (Yu et al., 2020). Numerous studies have shown that ethylene plays a crucial role in the regulation of diverse stress responses, including it to salt stress (Riyazuddin et al., 2020; Zhao et al., 2021a). In plants, ethylene is biosynthesized from S-adenosyl-L-methionine (SAM), which is synthesized from l-methionine to ATP catalyzed by S-adenosyl-L-methionine synthetases (SAMSs). SAM is conversed to 1-aminocyclopropane-1-carboxylicd (ACC) by ACC synthase (ACS). ACC is further oxygenated by ACC oxidase (ACO) to produce ethylene (Pattyn et al., 2021). The roles of ethylene in the regulation of salt tolerance are complex. Several studies have demonstrated that the overproduction of endogenous ethylene increases salinity stress tolerance in plants, while the inhibition of ethylene signaling leads to increased sensitivity to salinity stress (Zhang et al., 2011; Yang et al., 2013; Peng et al., 2014). For example, the loss-of-function mutant *eto1*, which exhibits increased levels of ethylene, shows enhanced salt tolerance (Jiang et al., 2013). Meanwhile, the *Arabidopsis* ethylene-insensitive mutants *ein2-5* and *ein3-1eil1-1* have been shown to exhibit salt-sensitive phenotypes (Lei et al., 2011; Peng et al., 2014). Moreover, ethylene has been found to be an essential positive mediator of salinity stress tolerance in rice. For example, the overexpression of acireductone dioxygenase, a metal-binding metalloenzyme encoded by *OsARD1*, elevates the endogenous ethylene release rate to reduce the sensitivity of rice plants to drought, salt, and osmotic stresses (Liang et al., 2019). These reports collectively suggest an important role for ethylene in salt stress tolerance in plants.

Receptor-like kinases (RLKs) play important roles in plant growth and development as well as in plant responses to biotic and abiotic stresses (Gao and Xue, 2012). Wall-associated kinases (WAKs) are a unique class of RLKs named for their close association with the plant cell wall (Verica and He, 2002). WAKs contain an extracellular domain that can be linked to molecules in the cell wall; this domain receives exogenous stimuli and then transfers them through the plasma membrane *via* a cytoplasmic serine/threonine kinase domain to initiate a downstream signaling cascade (Kohorn and Kohorn, 2012). The *Arabidopsis* genome encodes 5 WAKs and 21 WAK-like (WAKL) proteins. There are 5-fold more WAK members in rice than in *Arabidopsis*, indicating expansion of the WAK family in monocots (Zhang et al., 2005).

WAKs are involved in many physiological processes in plants (Kohorn and Kohorn, 2012; Kohorn, 2016). For instance, silencing of AtWAK2 results in small rosette leaves (Wagner and Kohorn, 2001), while the suppression of AtWAK4 produces a series of pleiotropic effects, including short primary roots, small rosette leaves, condensed inflorescence stems, unopened miniature flowers, and short siliques (Lally et al., 2001). In rice, silencing of the rice *indica OsiWAK1* gene results in dwarf seedlings, impaired root development, and sterility due to anther indehiscence, suggesting an important role in plant development *via* the regulation of cell expansion (Kanneganti and Gupta, 2011). Further, a loss of OsWAK91/OsDEES1 in rice plants can reduce fertility due to a defect in embryo development (Wang et al., 2012). These reports indicate that WAKs play essential roles in vegetative and reproductive development in plants. WAKs are also suggested to be involved in biotic stress responses. When the pathogen-responsive gene *AtWAK1* was knocked down, the expression of several pathogenesis-related genes was downregulated and the plants were more labile than wild type in response to a pathogen attack (He et al., 1998). The WAKL protein AtWAKL22/RFO1 is a novel type of dominant disease-resistance protein that mediates resistance to multiple *Fusarium* races (Diener and Ausubel, 2005). Another WAKL protein, AtWAKL10, is a positive regulator of basal immune responses and effector-triggered immunity in *Arabidopsis* (Bot et al., 2019). The first WAK identified in rice, OsWAK1, which is induced by *Magnaporthe oryzae*, plays a positive role in plant disease resistance (Li et al., 2009). Several other rice WAKs, such as OsWAK14, OsWAK91, OsWAK92, and OsWAK112, act as positive or negative regulators of quantitative resistance to the rice blast fungus (Delteil et al., 2016). In recent decades, the function of the *WAK* gene family in plant fungal disease resistance has also been characterized in maize, wheat, and tomato plants (Rosli et al., 2013; Hurni et al., 2015; Zuo et al., 2015; Saintenac et al., 2018). WAKs also help regulate plant adaptation to abiotic stresses. For instance, the mRNA and protein levels of AtWAK1 were found to rise quickly in response to aluminum treatment, and overexpression of *AtWAK1* retarded the growth inhibition of roots caused by aluminum stress in *Arabidopsis* (Sivaguru et al., 2003). Meanwhile, impaired expression of *AtWAKL4* increased plant hypersensitivity to excess K^+^, Na^+^, Cu^2+^, and Zn^2+^, while *WAKL4* overexpression conferred Ni^2+^ tolerance (Hou et al., 2005). More recently, AtWAKL10 was found to positively regulate salt stress while negatively regulating drought stress (Bot et al., 2019). In addition, WAK1 in tomato regulates leaf Na^+^ accumulation and alters the source–sink balance under high-salt conditions (Meco et al., 2020). Although these studies have deepened our understanding of plant WAKs, the molecular mechanisms underlying the functions of WAKs in salt stress responses are undefined, and the direct interacting components of WAKs in salt stress responses are unknown.

Rice, which is a staple food for more than half of the world’s population, faces various biotic and abiotic stresses during its three- to four-month growing season. Although 125 OsWAK genes have been annotated from rice genomes (Zhang et al., 2005), their functions are largely unknown and whether they are involved in salt stress responses are a mystery.

In this study, we investigated the function of OsWAK112 in salt stress responses. Our results show that OsWAK112 negatively regulates salt stress in rice depending on its kinase activity, possibly by inhibiting S-adenosyl-L-methionine synthase (SAMS) accumulation, which in turn decreases ethylene production.

## Materials and Methods

### Plant Materials and Constructs

The rice plants used in this study were *Oryza sativa japonica* (Jap). The *Arabidopsis thaliana* plants used were wild-type Columbia (Col).

To create 35S promoter-driven Myc-tagged OsWAK112 or OsWAK112^K678E^ or OsWAK112^D794A^ constructs, the OsWAK112 coding sequence was amplified by PCR with the primers OsWAK112-F and OsWAK112-R (Supplementary Table 1) to construct OsWAK112/pENTR using the commercial donor vector pENTR™/SD/D-TOPO™ (K242020; Invitrogen, Carlsbad, CA, United States). Next, OsWAK112^K678E^ and OsWAK112^D794A^ pENTR were generated using the Fast Mutagenesis System (FM111; TransGen Biotech, Beijing, China) with OsWAK112-K678E-F and OsWAK112-K678E-R or OsWAK112-D794A-F and OsWAK112-D794A-R as the overlapping mutagenic primers (Supplementary Table 1) and OsWAK112/pENTR as the template. Sequences encoding both native and mutated versions of OsWAK112 were cloned into the binary vector *pG7MH1* (Wang et al., 2013) using the Gateway LR Clonase™ II Enzyme Mix (11,791,020; Invitrogen).

To generate *OsWAK112* *RNAi* transgenic plants, a 581-bp *OsWAK112* fragment was amplified with specific primers OsWAK112RNAi-F and OsWAK112RNAi-R (Supplementary Table 1) using the LA Taq with GC Buffer kit (Takara, DRR20AG). The OsWAK112 RNAi fragment was inserted into the RNAi vector pTCK303 (Wang et al., 2004) through its KpnI/SpeI and BamHI/SacI sites.

For the native promoter-driven GUS construct proOsWAK112::GUS, the promoter sequence of OsWAK112 (with 2,108 base pairs upstream of the ATG) was amplified and inserted into the binary vector *pCAMBI1300::GUS* using *Hin*dIII and *Bam*HI.

For our bimolecular fluorescence complementation (BiFC) assays, the coding sequences of OsSAMS1/2/3 were amplified by PCR using the primers OsSAMS1/2/3-F and OsSAMS1/2/3-R (Supplementary Table 1) to construct OsSAMS1/2/3/pENTR. Then, the coding sequences of OsWAK112 and OsSAMS1/2/3 were individually introduced into 35S::X-NYFP and 35S::X-CCFP (Wang et al., 2013) using OsWAK112/pENTR and OsSAMS1/2/3/pENTR as templates.

For the co-immunoprecipitation (Co-IP) assays, the coding sequences of *OsSAMS1*, *OsSAMS2*, *OsSAMS3*, and the OsWAK112 kinase domain (OsWAK112KD) were cloned into *pMDC83* and *pG7MH1* to produce ectopic expression of OsSAMS1/2/3-GFP and OsWAK112KD-Myc, respectively.

The above-mentioned binary expression constructs were introduced into *Agrobacterium tumefaciens* strain EHA105 and then transformed into *Arabidopsis* or rice for stable transformation or co-infiltrated into 4-week-old *Nicotiana benthamiana* leaves for transient assays.

For prokaryotic protein expression, the coding sequence of the OsWAK112KD was amplified by PCR using the primers OsWAK112-KD-F and OsWAK112-KD-R (Supplementary Table 1) and inserted into pGEX-4T-1 to produce glutathione S-transferase (GST)-OsWAK112KD. Next, GST-OsWAK112^K678E^, GST-OsWAK112^K791R^, and GST-OsWAK112^D794A^ were generated using the Fast Mutagenesis System (FM111; TransGen Biotech) with OsWAK112-K678E-F/R, OsWAK112-K791R-F/R, or OsWAK112-D794A-F/R as the overlapping mutagenic primers (Supplementary Table 1) and GST-OsWAK112KD as the template. For myelin basic protein (MBP)-OsSAMS1/2/3, the coding sequences of OsSAMS1/2/3 were cloned into the destination vector gc-PML2C (Xin et al., 2021) using OsSAMS1/2/3/pENTR as the donor vector. The above-mentioned constructs were transformed into *Escherichia coli* strain BL21 for protein purification.

### Plant Growth and Salt Treatment Conditions

All rice plants were grown in a greenhouse with a 12-h/12-h light-dark cycle at 28°C (100μmolm^−2^ s^−1^ intensity) or in a paddy field under natural conditions (May to October in Hebei, China) for general growth and seed production. The *Arabidopsis* seedlings were grown either in a growth chamber under continuous light (100μmolm^−2^ s^−1^ intensity) or in a culture room under 16h of light/8h of darkness (90μmolm^−2^ s^−1^ intensity) at 22°C for seed production.

For salt sensitivity analyses of the rice in hydroponic culture, seeds were germinated in water for 2days, grown in a hydroponic culture solution (Ren et al., 2005) for 10days, and treated with a 200mM NaCl-containing culture solution for another 4days. After 8days of recovery, the survive rate was determined. To assess the salt response in solid medium, seeds were germinated on 0.5×Murashige and Skoog (MS) solid medium for 2days then transplanted to 0.5×MS solid medium without (0mM) or with 150mM or 200mM NaCl for 20days at 28°C in a light chamber under a 14-h/10-h light-dark cycle.

*Arabidopsis* seeds were sown on 0.5×MS medium with or without 125mM NaCl, kept for 2days in the dark at 4°C, grown under 16h of light/8h of darkness at 22°C for 10days, and then observed to determine the phenotype.

Seedlings that were still green and continuing to produce new leaves were registered as survivors. All experiments were repeated at least three times.

### Quantitative Semi-Quantitative Reverse Transcription Analysis

Total RNA from 7-day-old rice seedlings was isolated using TRIzol reagent (15596–026; Invitrogen) following the manufacturer’s instructions. cDNA was synthesized from 0.5 to 1μg of total RNA with a RevertAid First Strand cDNA Synthesis Kit (K1622; Thermo Fisher Scientific, Waltham, MA, United States) using oligo (dT)_16_ primer. To analyze *OsWAK112*, quantitative semi-quantitative reverse transcription (RT-qPCR) was performed using the SYBR Premix Ex Taq Reagent (RR420A; Takara, Otsu, Japan); the *Os18S* rRNA gene was used as an internal control. The primers used are listed in Supplementary Table 1.

### ROS Determination

The roots of 7-day-old rice plants treated with or without 150mM NaCl for 30min were stained with 5-(and-6)-chloromethyl-2ʹ,7ʹ-dichlorodihydrofluorescein diacetate, acetyl ester (CM-H_2_DCFDA; Thermo Fisher Scientific). The fluorescence intensity was determined with an Axio Imager (M2; Carl Zeiss, Jena, Germany); all pictures were analyzed using ImageJ. The fluorescent intensity of Jap at 0mM NaCl is set to 1.

### Subcellular Localization of OsWAK112

To investigate the subcellular localization of OsWAK112, the coding sequence of OsWAK112 from OsWAK112/pENTR was introduced into binary vector pMDC83 to construct the vector 35S:: OsWAK112-GFP, and 35S::GFP was used as a negative control vector. Both constructs were transferred to A. *tumefaciens* strain GV3101 and then transiently transformed into tobacco (*N. benthamiana*) leaves or transformed into onion (*Allium cepa*) epidermal cells by particle bombardment using the Bio-Rad PDS-1000/He system according to the manufacturer’s protocol. After 40 to 48h infiltration, localization of the protein was examined using a Zeiss LSM 510 confocal microscopy system with a 488nm laser for excitation from 500 to 515nm for GFP emission. 0.9M mannitol was used to induce plasmolysis.

### Kinase Assays

Recombinant GST-OsWAK112KD, GST-WAK112KD^K678E^, GST-WAK112KD^K791R^, and GST-WAK112KD^D794A^ were affinity purified using glutathione agarose beads (GE Healthcare, Chicago, IL, United States) and subjected to an *in vitro* kinase assay, which included incubation with 1μL of cold ATP (1mM) in kinase reaction buffer (50mM HEPES, 10mM MgCl_2_, 5mM MnCl_2_, and 1mM ATP, 10μCi of [γ-32P]ATP) at 30°C for 3h. The mixture was separated by 10% SDS-PAGE and detected with a Typhoon 9,410 imager (GE Healthcare).

### Protein–Protein Interaction Assays

For our BiFC assays, OsWAK112-NYFP and OsSAMSs-CCFP or YCE (control) were co-expressed in *N. benthamiana* leaves. Fluorescence from the BiFC signals was observed under a confocal microscope (Meta710-LCM; Carl Zeiss).

For the overlay assays, gel blots (polyvinylidene fluoride [PVDF] filters) containing GST-WAK112KD and GST were incubated with 2μg of MBP-OsSAMS1, MBP-OsSAMS2, and MBP-OsSAMS3, followed by anti-MBP-HRP (E8038S; New England BioLabs, Ipswich, MA, United States).

In our Co-IP assays, *N. benthamiana* leaves transiently co-expressing OsSAMSs-GFP and OsWAK112KD-Myc, or expressing OsSAMSs-GFP or OsWAK112KD-Myc alone, were harvested and extracted with IP buffer (50mM Tris-HCl, pH 7.5, 150mM NaCl, 2mM EDTA, and 0.1% NP-40) and then incubated with anti-GFP agarose at 4°C for 2h. Next, the proteins were washed with IP buffer five times and then boiled at 100°C in water for 5min with 2×loading buffer. The proteins were then separated by SDS-PAGE. Anti-Myc (019M4760V; Sigma-Aldrich, St. Louis, MO, United States) and anti-GFP antibodies (HT801-01; TransGen Biotech) were used to detect OsWAK112KD and OsSAMS1/2/3, respectively.

### Ethylene and SAMS Measurement

For ethylene measurement, 7-day-old wild-type (Jap) and *OsWAK112-*overexpressing (*OsWAK112OE*) rice seedlings treated with or without 200mM NaCl for 24h were placed into 15-mL vials containing 3ml of 0.5×MS medium and closed with a rubber cap on the top; the vials were then allowed to sit for 24h (16-h light/8-h dark) at 28°C. A total of 1mL of the air from the headspace of each vial was taken to inject into a gas chromatograph to measure the ethylene content.

For SAMS measurement, total protein from 7-day-old Jap and *OsWAK112OE* seedlings treated with or without 200mM NaCl for 24h was extracted and used for SAMS content measurement with a SAMS ELISA Kit (SY-P09712; Shanghai Win-Win Biotechnology Co., Ltd., Shanghai, China). The assay was performed according the manufacturer’s protocol.

### Western Blotting in Tobacco Leaves

To test whether OsWAK112 affects OsSAMSs protein stability, OsSAMS1-GFP alone or OsSAMS1 together with OsWAK112-Myc was filtrated in tobacco leave. After 40 to 48h, tobacco leave was separated into two parts, treated in liquid 1/2 MS medium with or without 200mM NaCl for 2h, and then, the total protein was extracted for Western blotting.

## Results

### *OsWAK112* Negatively Regulates Salt Responses in Rice

In this study, we examined the involvement of *OsWAK112* in plant salt responses. OsWAK112 bound directly to and inhibited OsSAMSs to suppress the induction of ethylene production so as to negatively regulate salt tolerance.

WAKs are protein kinases with important roles not only in plant growth and development but also in plant responses to exogenous stresses (Hou et al., 2005; Bot et al., 2019; Meco et al., 2020); however, their role in salt stress responses is poorly understood. Published microarray data revealed that the expression levels of several WAKs are regulated by salt (Mishra et al., 2018). Herein, the expression pattern of one of them, *OsWAK112* (*Os10g10130*; Mishra et al., 2018), was examined under high-salt conditions. Its transcript level decreased gradually as the treatment time increased; at 24h, it was reduced to nearly half of the control level (Supplementary Figure S1), indicating that OsWAK112 is a potential salt-related regulator in rice.

**Figure 1:**
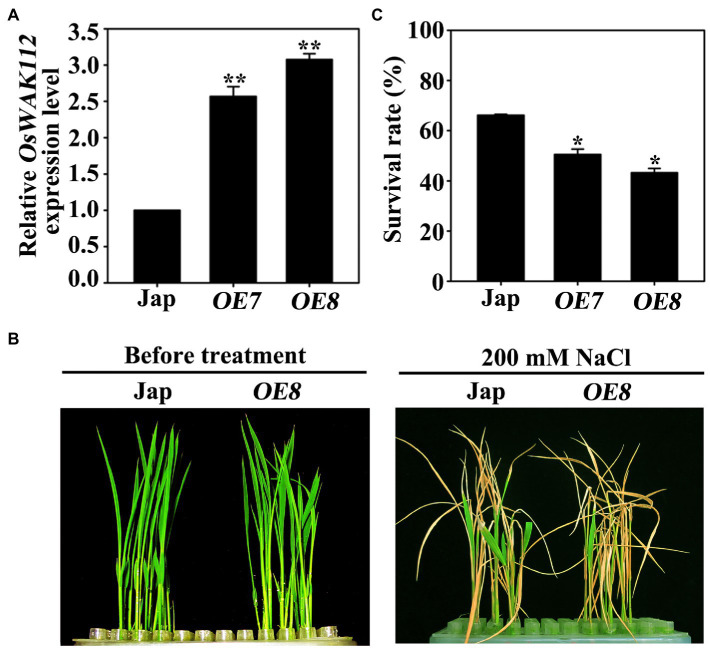
Phenotypes of the *OsWAK112OE* lines under salt stress. **(A)** The *OsWAK112* transcript levels in 7-day-old Jap and *OsWAK112OE* lines as determined by RT-qPCR. **(B)** A comparison of the phenotypes of Jap plants and a representative *OsWAK112OE* line (*OE8*) before and after salt treatment. Ten-day-old seedlings were used as described in the Materials and Methods. **(C)** Survival rates of Jap and two individual *OsWAK112OE* lines following salt exposure. In (A,C), each data point represents the mean ± standard error (SE) from three biological repeats (*n*≥50). Asterisks indicate a difference relative to Jap (Student’s *t*-test, ^*^p<0.05 and ^**^p<0.01).

To determine the biological function of *OsWAK112* in salt responses, ectopic expression lines using a 35S promoter-driven *OsWAK112* coding sequence with a Myc tag were generated in a wild-type Jap background. Two independent *OsWAK112OE* lines named *OE7* and *OE8* were used in our subsequent experiments because they exhibited high transcript levels (approximately 2.6- or 3.2-fold that in Jap; Figure 1A). Under normal growth conditions, the *OsWAK112OE* plants exhibited no clear difference from Jap plants (Figure 1B, before treatment). However, after being treated with 200mM NaCl for 4days and subsequently allowed to recover in regular hydroponic culture for another 8days, the *OsWAK112OE* plants were drier and yellower than the Jap plants (Figure 1B), in accordance with their lower survival rates (50% for *OE7* and 43% for *OE8*) compared to Jap (63%) (Figure 1C), indicating that *OsWAK112* induces poor salt resistance in rice.

**Figure 2:**
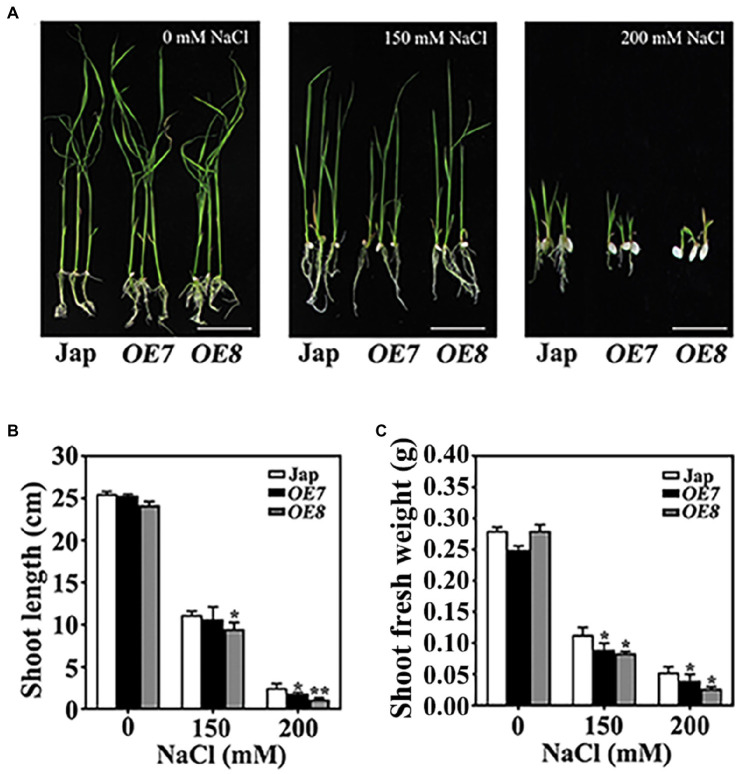
*OsWAK112* overexpression confers salt hypersensitivity in rice. **(A)** The performance of *OsWAK112OE* seedlings compared with Jap on media containing the indicated concentrations of NaCl. Scale bar=2cm. The shoot lengths **(B)** and fresh shoot weights **(C)** of 10-day-old seedlings were analyzed. The experiments were repeated three times. Each data point represents the mean±SE (*n*≥10). Asterisks indicate a difference relative to Jap (Student’s *t*-test, ^*^p<0.05 and ^**^p<0.01) in **(B,C)**.

To confirm the function of OsWAK112 in plant salt tolerance, the salt responses of *OE7* and *OE8* plants were examined on solid medium. After 2 days of germination on regular medium, seeds were transplanted into medium containing 0, 150, or 200mM NaCl. The *OE7* and *OE8* seedlings did not show any difference from Jap seedlings when grown on regular medium (Figure 2A, 0mM NaCl); however, they showed greater growth inhibition by salt than Jap plants depending on the NaCl concentration (Figure 2A). The shoot length and shoot fresh weight of *OE7* and *OE8* were both lower than those of Jap (Figures 2B,C).

To completely investigate the function of OsWAK112, the RNAi of *OsWAK112* transgenic rice plants was also generated by introducing the vector pTCK303. Two independent OsWAK112 RNAi lines (RNAi1 and RNAi3, which showed around 4 and 7% of *OsWAK112* mRNA level) were assessed under salt stress conditions. Without NaCl treatment, the OsWAK112 RNAi plants were indistinguishable from wild-type japonica (Jap; Supplementary Figure S2). After treatment with 200mM NaCl for 4 days and subsequent growth by regular hydroponic culture for 8 days, the RNAi plants showed significantly higher survival rates compared with Jap (Supplementary Figure S2).

Taken together, these findings (Figures 1, 2 and Supplementary Figure S2) demonstrate that *OsWAK112* plays a negative role in salt tolerance.

### OsWAK112 Positively Regulates Hydrogen Peroxide (H_2_O_2_) Accumulation in Plants Under High-Salt Conditions

Salt stress induces the rapid accumulation of ROS, and an imbalance in ROS levels often triggers serious membrane damage in plants (You and Chan, 2015; Yang and Guo, 2018). To examine whether the increased salt sensitivity of *OsWAK112OE* was related to the ROS level, the quantity of H_2_O_2_, a major and stable ROS, was evaluated using CM-H_2_DCFDA with 7-day-old roots from Jap and *OsWAK112OE* lines with or without 200mM NaCl treatment. This probe can be transported into cells, where its acetate groups are passively cleaved by intracellular esterases, producing the fluorescent compound dichlorodihydrofluorescein (DCF; Wang et al., 2021). As shown in Supplementary Figure S3A, without salt treatment, the H_2_O_2_ levels in the *OsWAK112OE* lines were slightly lower than that in Jap. After salt treatment, the H_2_O_2_ levels in Jap and in the two *OsWAK112OE* lines were dramatically increased, indicating the induction of H_2_O_2_ production by salt as described previously (Li et al., 2014). However, the H_2_O_2_ levels in the *OsWAK112OE* lines were significantly higher (110 and 103% higher than in *OE7* and *OE8* [individual controls], respectively) than in Jap (46% higher than its control; Supplementary Figures S3A,B), revealing that the *OsWAK112OE* plants accumulated more ROS during salt treatment.

### OsWAK112 Is Mainly Located in the Plasma Membrane and Is Associated With the Cell Wall

To investigate the tissue-specific expression pattern of *OsWAK112*, the 2,108-base pair promoter sequence was inserted into a pCAMBIA1300 vector harboring the gene encoding GUS. This *proOsWAK112::GUS* construct was introduced into rice plants by *A. tumefaciens*-mediated callus transformation. Histochemical staining revealed *OsWAK112* expression in coleoptiles (Supplementary Figure S4A), ligules (Supplementary Figure S4B), leaf blades (Supplementary Figure S4C), stems (Supplementary Figures S4D,E), roots (Supplementary Figure S4F), and the radicles of germinated seeds (Supplementary Figure S4G) during vegetative growth. During reproductive growth, *OsWAK112* was observed in paleae (Supplementary Figure S4H), stamens, and stigmas (Supplementary Figure S4I). These results show that *OsWAK112* is expressed in most plant tissues.

**Figure 3:**
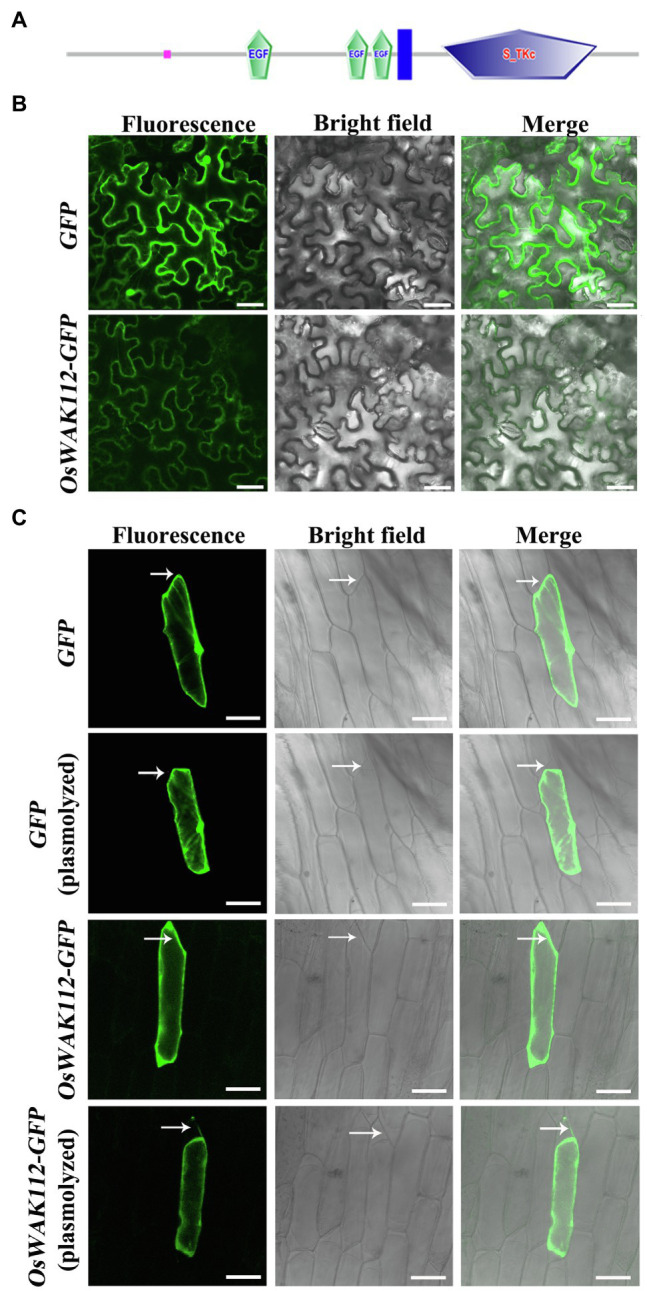
The subcellular localization of *OsWAK112*. **(A)** The protein structure of OsWAK112 as predicted by SMART. Pink box: low complexity region. EGF: epidermal growth factor-like domain. Blue box: transmembrane region. S-TKc: serine/threonine protein kinase, catalytic domain. (B,C) Subcellular localization of OsWAK112-GFP in tobacco and onion epidermal cells. Arrows in **(C)** indicate the cell wall region where plasmolysis happened. Scale bar=50μm in **(B)** and 100μm in (C).

Online sequence analyses have shown that OsWAK112 possesses a typical receptor kinase structure, including an extracellular domain, a transmembrane domain, and an intracellular kinase domain.^1^ Thus, it is speculated to be a plasma membrane protein (Figure 3A); however, the sub-location of OsWAK112 is unclear. Thus, the full-length coding sequence of *OsWAK112* fused with the sequence encoding green fluorescence protein (GFP) was generated and expressed transiently in tobacco (*N. benthamiana*) and onion (*A. cepa*) epidermal cells. As shown in Figure 3, GFP alone was expressed ubiquitously in the intracellular region of the tobacco epidermal cells (Figure 3B, GFP) and onion epidermal cells (Figure 3C, GFP), whereas OsWAK112-GFP was detected only in the region of the plasma membrane (Figures 3B,C, OsWAK112-GFP). To verify whether OsWAK112 is associated with the cell wall, onion epidermal cells were treated with 0.9 M mannitol to induce plasmolysis. After plasmolysis, OsWAK112-GFP was detected in the plasma membrane region and in the cell wall region (Figure 3C, OsWAK112-GFP plasmolyzed). In contrast, the control protein (GFP) was detected only in the intracellular region (Figure 3C, GFP plasmolyzed). These data indicate that OsWAK112 is localized to the plasma membrane and that it may connect tightly to the cell wall. Moreover, these data suggest that OsWAK112 is a wall-associated protein in plant cells.

### The Kinase Activity of OsWAK112 Plays an Important Role in Salt Stress

To investigate the function of OsWAK112 further, we performed a sequence alignment and homology analysis between OsWAK112 and multiple RLKs in *Arabidopsis* using GenBank from the National Center for Biotechnology Information.^2^ The sequence of OsWAK112 showed 40–60% similarity with those of WAK and cytosolic receptor kinase family members in *Arabidopsis*, and most of the conserved sequences were in the kinase domain (Supplementary Figure S5).

**Figure 4:**
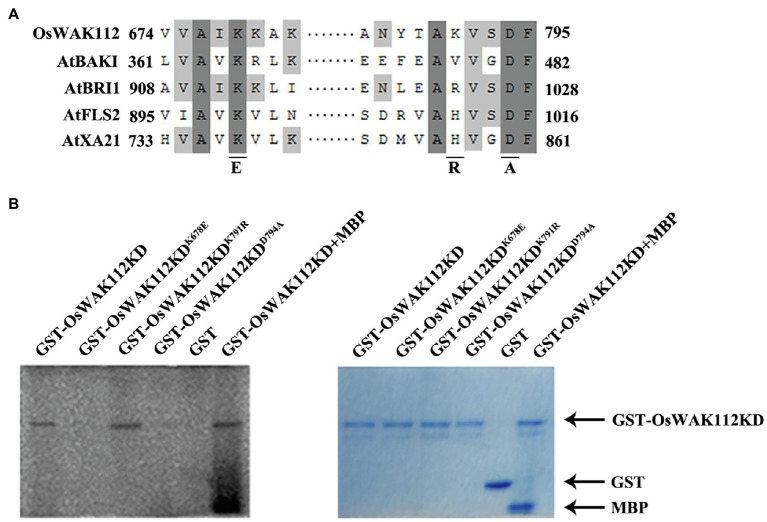
OsWAK112 possesses kinase activity. **(A)** Alignment of a conserved motif from the OsWAK112KD with that from other known receptor-like kinases (RLKs). Underlines indicate conserved residues in the active kinase that were mutated to the indicated amino acid in the following experiment. **(B)** GST-OsWAK112KD showed autophosphorylation and substrate (MBP) phosphorylation. The mutated form GST-WAK112KD^K791R^ also showed autophosphorylation, whereas the mutated forms GST-OsWAK112KD^K678E^ and GST-OsWAK112KD^D794A^ did not. The left panel shows autoradiography and the right panel Coomassie blue (CBB) staining of the gel.

The sequence alignment of the kinase domain from OsWAK112 with those from several kinases in *Arabidopsis* is shown in Figure 4A. A typical ATP-binding motif and kinase active site were located in the intracellular domain of OsWAK112 (Figure 4A), indicating that it is an active kinase. Although it is predicted that OsWAK112 is linked to the cell wall *via* its cytosolic kinase domain (Figure 4A), whether WAK112 has kinase activity is unknown. To test this, the lysine (Lys-678) in the ATP-binding region and lysine (Lys-791) and aspartic acid (Asp794) in the activation loop of the kinase domain (KD) were mutated to generate WAK112KD^K678E^, WAK112KD^K791R^, and WAK112KD^D794A^, respectively (Figure 4B). The wild-type and mutated forms of the WAK112 KD were produced as fusions with GST. An *in vitro* kinase assay showed that the OsWAK112KD produced strong autophosphorylation and phosphorylation of MBP (Figure 4B, lanes 1 and 6). WAK112KD^K791R^ also showed strong autophosphorylation (Figure 4B, lane 3), but the mutated forms (GST-OsWAK112^K678E^ and GST-OsWAK112^D794A^; Figure 4B, lanes 2 and 4) and GST alone (Figure 4B, lane 5) did not exhibit autophosphorylation activity, indicating that OsWAK112 is an active RLK and that both Lys-678 and Asp-794 are necessary for its activity.

**Figure 5:**
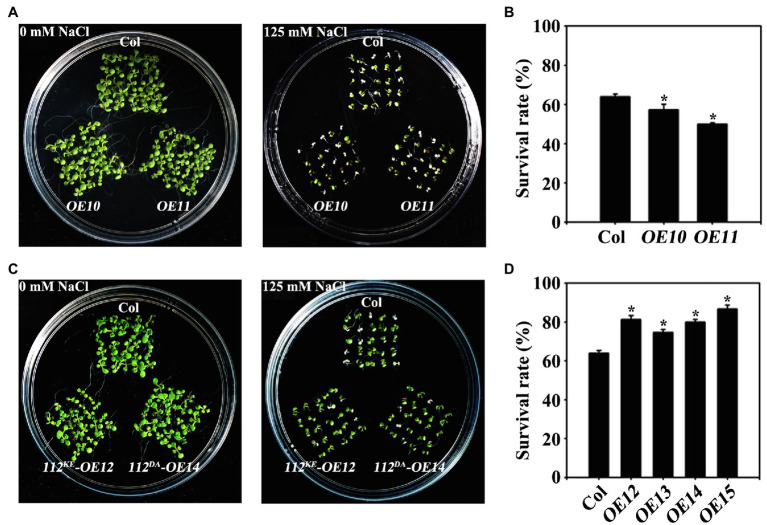
OsWAK112 mediates salt sensitivity depending on its kinase activity. *Arabidopsis* plants overexpressing *OsWAK112* showed hypersensitivity to salt compared with Col **(A,B)**, while A*rabidopsis* plants overexpressing *OsWAK112*^K678E^ and *OsWAK112*^D794A^ showed hyposensitivity to salt compared with Col **(C,D)**. The indicated seedlings were grown on 0.5×MS medium supplemented with or without 125mM NaCl for 10days, and the salt survival rate was calculated. *OE10* and *OE11* represent two independent *35S:OsWAK112-Myc/Col* transgenic lines. *OE12* and *OE13* represent two independent *35S:OsWAK112^K678E^-Myc/Col* transgenic lines. *OE14* and *OE15* represent two independent *35S:OsWAK112^D794A^-Myc/Col* transgenic lines. The experiments were repeated three times. Each data point represents the mean ± SE (*n*≥25). Asterisks indicate a difference relative to Col (Student’s *t*-test, ^*^p<0.05) in **(B-D)**.

To assess the contribution of the kinase activity of OsWAK112 to plant salt sensitivity, we generated transgenic *Arabidopsis* expressing OsWAK112, OsWAK112^K678E^, and OsWAK112^D794A^ as Myc fusions under the control of the 35S promoter. Western blotting showed that wild-type and mutated forms of OsWAK112 were well expressed in *Arabidopsis* (Supplementary Figure S6). On regular medium, little difference was noted among the seedlings (Figures 5A,C, 0mM NaCl). On 0.5×MS medium containing 125mM NaCl, the WAK112-overexpressing seedlings (*OE10* and *OE11*) exhibited more strongly bleached cotyledons (Figure 5A) and lower survival rates than wild-type Col seedlings (57% for *OE10* and 50% for *OE11*, compared with 63% for Col; Figure 5B). This is consistent with the effects of overexpressed *OsWAK112* on salt hypersensitivity in rice (Figures 1, 2). However, the *Arabidopsis* 35S::OsWAK112^K678E^ (named *OE12* and *OE13*) and 35S::OsWAK112^D794A^ (named *OE14* and *OE15*) seedlings showed less damage in response to salt exposure, with around 70–86% survival (higher than that of Col; Figures 5C,D). These results suggest that the kinase activity of OsWAK112 affects salt resistance as a negative regulator.

### OsWAK112 Interacts With OsSAMS1/2/3 and Affects Ethylene Production Under Salt Stress

Our work indicated that OsWAK112 plays a negative role in salt tolerance. As a wall-associated protein, it is likely that OsWAK112 confers salt response by directly binding to specific target proteins. Then, we sought to identify its interacting proteins to gain insight into the roles of OsWAK112 in salt response.

**Figure 6:**
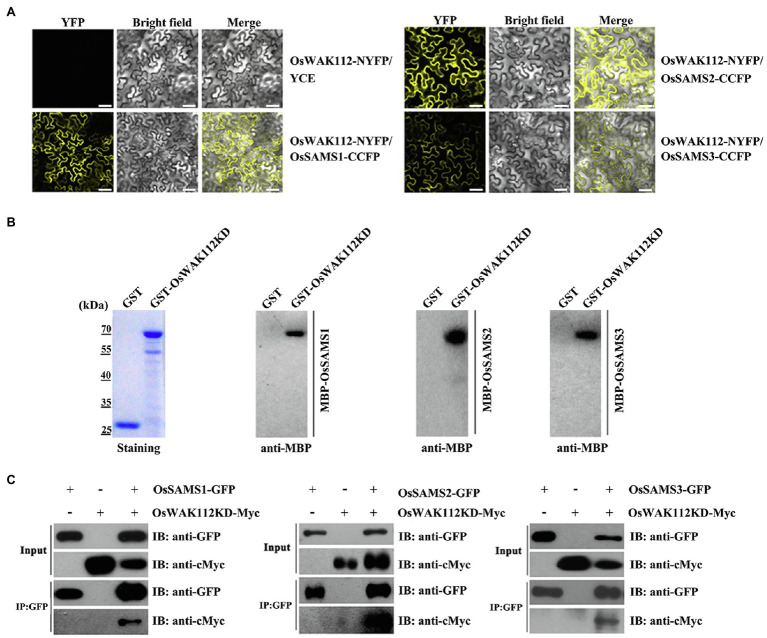
OsWAK112 interacts with OsSAMS1, OsSAMS2, and OsSAMS3 *in vitro* and *in vivo*. **(A)** MBP-OsSAMSs bound to the GST-WAK112KD fusion but not to GST in a gel blot overlay assay. Coomassie blue staining in the left panel indicates the loading of GST-WAK112KD and GST. Bound proteins were probed with anti-MBP-HRP antibodies. OsWAK112 interacted with OsSAMSs in BiFC assays **(B)** and Co-IP assays **(C)** in transiently transformed *N. benthamiana* leaves. Scale bar=50μm in **(B)**.

According to a structural analysis conducted using WebLab ViewerLite (Accelrys, San Diego, CA), several salt-associated proteins harbor important binding elements for OsWAK112, including GRP1, GRP2, GRP3, OsRP-1, PSBP1, and SAMSs (without OsACS and OsACO) as WAK112 candidate interacting proteins. Firstly, we performed BiFC assay to test whether OsWAK112 interacted with these proteins. The sequences encoding GRP1, GRP2, GRP3, OsRP-1, OsPSBP1, SAMS1/2/3, and OsWAK112 were fused with those sequences encoding the N-terminal half and C-terminal half of YFP to generate GRPs-CCFP, OsRP-1-CCFP, OsPSBP1-CCFP, OsSAMSs-CCFP, and OsWAK112-NYFP, respectively. The constructs were then co-infiltrated into *N. benthamiana* leaves. When the OsGRP1, OsGRP2, OsGRP3, OsRP-1, and OsPSB1 fusion with CCFP co-filtrated in OsWAK112 fusion with NYFP into *N. benthamiana* leaves, no fluorescence signal was observed (data not shown). However, co-expression of OsSAMS1/2/3-CCFP and OsWAK112-NYFP resulted in a strong YFP signal, while the negative control (OsWAK112-NYFP+YCE) produced almost no signal (Figure 6A), demonstrating the specific interaction of OsWAK112 with OsSAMSs *in vivo*. The direct interaction between OsWAK112 and OsSAMS1/2/3 was then examined in overlay assays. Purified MBP-OsSAMS1, MBP-OsSAMS2, and MBP-OsSAMS3 were used as primary antibodies and incubated with PVDF membranes containing GST and GST-OsWAK112KD; MBP-HRP antibodies were used as secondary antibodies. MBP-OsSAMS1, MBP-OsSAMS2, and MBP-SAMS3 were detected at positions corresponding to the size of GST-OsWAK112KD, but no band was detected at the position of the GST tag, suggesting a direct interaction between OsWAK112 and OsSAMS1/2/3 *in vitro* (Figure 6B). In addition, the overlay assay showed that D794A mutation in OsWAK112 did not affect its interaction with OsSAMS1 (Supplementary Figure S7), indicating the conserved aspartic acid or kinase activity was not required for this interaction. Furthermore, their interaction was further tested using Co-IP assays. The sequences encoding OsSAMS1/2/3 and OsWAK112KD were fused with GFP and Myc to generate OsSAMSs-GFP and OsWAK112KD-Myc, respectively. The constructs were then co-infiltrated into *N. benthamiana* leaves. Co-IP was achieved using *N. benthamiana* leaves transiently expressing OsSAMSs-GFP and OsWAK112KD-Myc. OsWAK112KD-Myc could be co-precipitated only when OsSAMSs-GFP was present (Figure 6C), confirming the interaction between OsWAK112 and OsSAMS1, OsSAMS2, or OsSAMS3 *in vivo*.

Together, these results suggest that OsWAK112 interacts physically with OsSAMS1/2/3 *in vitro* and *in vivo*. Next, the biological function of the OsWAK112–OsSAMS1 interaction was investigated in tobacco plants. A co-infiltration assay showed that OsSAMS1-GFP was well expressed in tobacco. Compared with mock treatment, 2h of treatment with 200mM NaCl decreased the levels of OsSAMS1 when co-expressed with OsWAK112, indicating that OsWAK112 affects OsSAMS1 stability under saline conditions (Supplementary Figure S8). Together, these data suggest that OsWAK112 interacts with OsSAMS1/2/3 and promotes OsSAMS1 degradation in the presence of salt.

**Figure 7:**
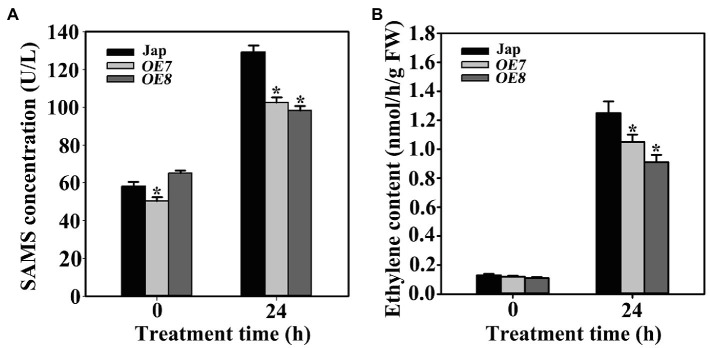
OsWAK112 affects ethylene production under salt stress. **(A)** The amount of ethylene per gram fresh weight in 7-day-old Jap, *OE7*, and *OE8* seedlings grown under normal conditions or treated with 200mM NaCl for 24h. **(B)** The SAMS contents of 7-day-old Jap, *OE7*, and *OE8* seedlings grown under normal conditions or treated with 200mM NaCl for 24h. In **(A,B)**, Student’s *t-*tests were conducted using data from transgenic seedlings compared to those from Jap. Asterisks indicate a significant difference at *p*<0.05.

To investigate the influence of OsWAK112 on OsSAMS contents in transgenic plants, the SAMS contents were measured in *OE7*, *OE8*, and Jap plants. The data showed that SAMS contents were not significantly different between Jap and *OE7/8* under normal conditions, but the SAMS levels were lower in *OE7* and *OE8* than in Jap following salt treatment (Figure 7A).

As a major abiotic stress, salt can trigger the production of stress-related hormones and initiate endogenous hormone signaling, resulting in plant resistance reactions (Zhao et al., 2021b). Ethylene is a stress hormone closely related with salt stress (Riyazuddin et al., 2020). In plants, ethylene is biosynthesized from S-adenosyl-L-methionine (SAM; Zhao et al., 2021a), which is synthesized from l-methionine and ATP catalyzed by SAMSs. To explore the mechanism underlying the function of OsWAK112 in plant salt responses, the ethylene content was measured in Jap and *OsWAK112OE* plants. Under normal growth conditions, the ethylene contents did not differ much between Jap and the *OsWAK112OE* lines. After salt treatment, the ethylene contents increased in all of the plants; however, they were lower in *OE7* and *OE8* than in Jap (Figure 7B), while they were higher in *RNAi1* and *RNAi3* than in Jap (Supplementary Figure S9). Together, these data suggest that OsWAK112 interacts with OsSAMS1/2/3 and promotes OsSAMS1 degradation, resulting in decreased ethylene production in the presence of salt.

## Discussion

Soil salinity is a significant threat to the growth, productivity, and quality of crop plants. During plant salt stress responses, multiple signaling cascade components are activated. RLKs have previously been shown to be involved in salt responses (de Lorenzo et al., 2009; Ouyang et al., 2010; Lin et al., 2020; Passricha et al., 2020; Yuchun et al., 2021); however, the function of a unique group of RLKs, known as WAKs, in salt tolerance remains unclear. WAKs likely associate with pectin in the cell wall and are necessary for both cell expansion during development and mediation of the response to stresses (Kanneganti and Gupta, 2011; Wang et al., 2012; Delteil et al., 2016). In the present study, we found that *OsWAK112* functions as a kinase to negatively mediate plant salt responses by reducing the ethylene content *via* a direct interaction with OsSAMS1.

### OsWAK112 Negatively Mediates Salt Sensitivity

To our knowledge, OsWAK112 has only been shown to play a negative role in biotic stress tolerance in rice (Delteil et al., 2016); there are no reports of its function in response to abiotic stresses. The time-dependent suppression of *OsWAK112* by NaCl treatment (Supplementary Figure S1 [Supplementary-material SM1]) suggests that OsWAK112 is closely related to plant responses to high salinity. When *OsWAK112* was overexpressed in rice, two independent lines (*OE7* and *OE8*) showed hypersensitivity to salt stress. Further, the higher *OsWAK112* expression level and the lower the survival rate in *OE8* and *OE7* (Figures 1, 2), and the less OsWAK112 expression level and the higher survival rate in RNAi lines compared with Jap (Supplementary Figure S2), indicate that the *OsWAK112* expression level correlates negatively with plant salt resistance. These findings from morphological and physiological measurements indicate that OsWAK112 is a negative regulator of salt resistance reactions in rice.

Salinity stress induces ROS accumulation. Low levels of ROS usually act as signal molecules in salt signaling networks, but excessive accumulation of ROS is highly toxic to proteins, lipids, and nucleic acids, resulting in cell death (You and Chan, 2015). H_2_O_2_ is an especially stable ROS in plant cells. We found that more H_2_O_2_ accumulated in *OsWAK112OE* (*OE7/8*) plants under salt stress and that the variation in their H_2_O_2_ levels was consistent with that of their *OsWAK112* expression levels (Supplementary Figure S3). Excessive accumulation of H_2_O_2_ accompanied by strong *OsWAK112* expression under conditions of salt stress may damage plant cells, resulting in reduced survival.

### OsWAK112 Relays Salt Stress Signals Through Kinase Activation

As a kinase, how does OsWAK112 coordinately regulate plant responses to salt stress? It would be a worthwhile question to investigate.

Each WAK protein contains an extracellular calcium-binding domain, a transmembrane domain, and a cytoplasmic serine/threonine kinase domain (Kohorn and Kohorn, 2012). The extracellular domain senses the signal, while the cytosolic protein kinase domain translates the signal to downstream components *via* phosphorylation. OsWAK112 contains an arginine residue in its kinase catalytic motif (RDxxxxN) and is classified as a WAK-RD kinase (de Oliveira et al., 2014). Several RLKs have been shown to be involved in salt responses depending on their kinase activity. For example, SIT1, a lectin RLK, negatively regulates salt responses in rice, and it kinase activity plays an important role in its salt response (Li et al., 2014). Another receptor-like cytoplasmic kinase, STRK1, phosphorylates and stimulates the activity of catalase C to adjust H_2_O_2_ concentrations and improve salt tolerance (Zhou et al., 2018). Herein, we used an *in vitro* kinase assay to show that OsWAK112 has autophosphorylation activity and strong substrate activity (Figure 4). We found that Lys-791 near the DFG motif of kinase domain VII produced the same autophosphorylation signal as wild-type OsWAK112 *in vitro* (Figure 4); therefore, it appears that Lys-791 is not essential for the kinase activity of the enzyme *in vitro*. That may be because Lys-791 is not conserved in OsWAK112 compared with other known kinases. Our results show that OsWAK112 exhibited kinase activity depending on its conserved residues Lys-678 and Asp-794 (Figure 4). Lys-678 is in the AXK motif of kinase domain II for interaction with the phosphates of ATP. Asp-794 in the DFG motif of kinase domain VII chelates a Mg^2+^ ion to orient the γ-phosphate of ATP for transfer (Hanks and Hunter, 1995). When Lys-678 or Asp-794 was mutated, OsWAK112 lost its kinase activity (Figure 4). Exogenous expression of *OsWAK112* in *Arabidopsis* made the transgenic plants hypersensitive to salt (Figure 5), indicating that *OsWAK112* functions as a negative regulator of salt responses in both *Arabidopsis* and rice. However, exogenous overexpression of *OsWAK112^K678E^* or *OsWAK112^D794A^* did not increase the sensitivity of *Arabidopsis* to salt (Figure 5). A plausible explanation is that kinase-dead forms of *OsWAK112* could still sense the salt stimulus and bind to downstream substrates, but the signal could not be transduced due to the loss of kinase activity (Figure 4), resulting in a dominant-negative phenotype. For example, in plants, when ERECTA lack the cytoplasmic kinase domain it acts as dominant-negative receptors by blocking the normal activity of the endogenous counterparts (Shpak et al., 2003). And in plant hormone brassinosteroids signaling pathway, the receptor kinase BRI1 phosphorylates the substrate BSK3 on S215. When S215 mutated into A, S215A had a dominant-negative effect: Plants overexpressing the S215A version of OsBSK3 were smaller and dwarfed than the nontransgenic controls when grown in the light under the same conditions (Zhang et al., 2016).

### OsWAK112 Mediates Salt Sensitivity by Affecting Ethylene Homeostasis

Ethylene homeostasis is tightly controlled to maintain its dual functions in growth inhibition and growth stimulation. Under normal conditions, the ethylene concentration is low and only increases dramatically at defined developmental stages, such as fruit ripening (Zhao et al., 2021a). A variety of environmental stimuli, including salt stress, can also induce ethylene production (Nascimento et al., 2018; Riyazuddin et al., 2020), in which the ACC synthase-induced conversion of SAM to ACC is a key step. SAM is synthesized by SAMSs, which play important roles in regulating plant–environment interactions. Studies show that SAMSs can be induced by multiple stress treatments and that they play important roles in regulating plant tolerance to environmental changes, especially soil salinity and drought. For example, in sugar beet, *BvM14-SAMS2* is induced by salt, and its ectopic expression in *Arabidopsis* plants increased salt and oxidation resistance (Ma et al., 2017). In tobacco, overexpression of *SlSAMS1* affects ethylene emission and improves drought and salt stress tolerance (Zhang et al., 2020). We found that OsWAK112 interacted directly with OsSAMS1/2/3 in overlay, BiFC, and Co-IP assays (Figure 6). Among these three SAMSs in rice, OsSAMS1 plays a predominant role and is expressed abundantly in every tissue (Li et al., 2011). We found that OsWAK112 promoted OsSAMS1 degradation under high-salt conditions (Supplementary Figure S8). There was a lower OsSAMS content and a reduced ethylene content in our *OsWAK112OE* plants, following salt treatment (Figure 7), while a higher ethylene existed in OsWAK112 RNAi plants content under salt stress. Consistent with our study, OsARD1, a metal-binding metalloenzyme involved in the methionine salvage pathway, enhances ethylene biosynthesis and reduces the salt sensitivity of rice (Liang et al., 2019). These data suggest that OsWAK112 is an active kinase that interacts with OsSAMS1/2/3, promoting OsSAMS1 degradation, and reducing ethylene production under saline conditions.

Soil salinity is a serious threat to crop growth and agricultural productivity. Our findings demonstrate that salt sensitivity in rice is mediated by OsWAK112 depending on its kinase activity. These findings provide important new information for engineering salt-tolerant crops.

#### Accession Numbers

Sequence data from this article can be found in GenBank/EMBL under the following accession numbers: *OsWAK112* (Os10g10130), *OsSAMS1* (Os05g04510), *OsSAMS2* (Os01g22010), and *OsSAMS3* (Os01g18860).

## Data Availability Statement

The original contributions presented in the study are included in the article/Supplementary Material, and further inquiries can be directed to the corresponding authors.

## Author Contributions

LZ conceived the project and designed the research. LL and YW carried out most of the experiments, phenotypic observations, RT-qPCR analysis, the rice and *Arabidopsis* transgenic experiments, and Western blot analysis and data analysis. XL participated in the data analysis. LZ and J-XS wrote the article with contributions from all authors and revised and proofread the manuscript. All authors participated in revising the manuscript and approved the final version.

## Funding

This work was supported by grants from the National Natural Science Foundation of China (31770297 to LZ and 31872830 to J-XS) and the Department of Education of Hebei Province (BJ2019025 to J-XS).

## Conflict of Interest

The authors declare that the research was conducted in the absence of any commercial or financial relationships that could be construed as a potential conflict of interest.

## Publisher’s Note

All claims expressed in this article are solely those of the authors and do not necessarily represent those of their affiliated organizations, or those of the publisher, the editors and the reviewers. Any product that may be evaluated in this article, or claim that may be made by its manufacturer, is not guaranteed or endorsed by the publisher.

## Footnotes

^1^ http://smart.embl.de

^2^ http://www.ncbi.nlm.nih.gov
