# Supplementary material for: OsWAK112, A Wall-Associated Kinase, Negatively Regulates Salt Stress Responses by Inhibiting Ethylene Production
Source: Front Plant Sci. 2021 Oct 5;12:751965. doi: 10.3389/fpls.2021.751965 (PMC8523997; doi:10.3389/fpls.2021.751965)
Supplement: Supplementary file 1 [file Data_Sheet_1.pdf]

Supplementary Material

1.1 Supplementary Figures

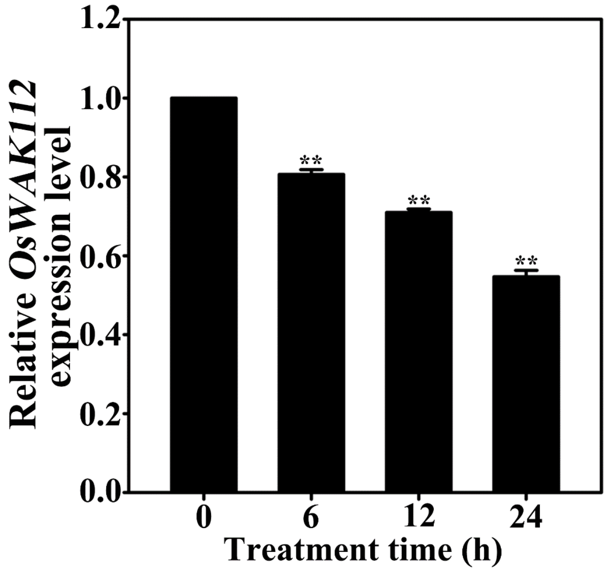

Supplementary Figure S1. OsWAK112 expression is repressed by salt.

Seven-day-old rice seedlings were treated with 200 mM NaCl, and tissues were collected at the indicated time points. The transcript level of *OsWAK112* was detected by RT-qPCR. *Os18S* was used as an internal reference. Values are presented as the means of replicates ± SE. Data were analyzed by Student's *t*-test. ***P* < 0.01.

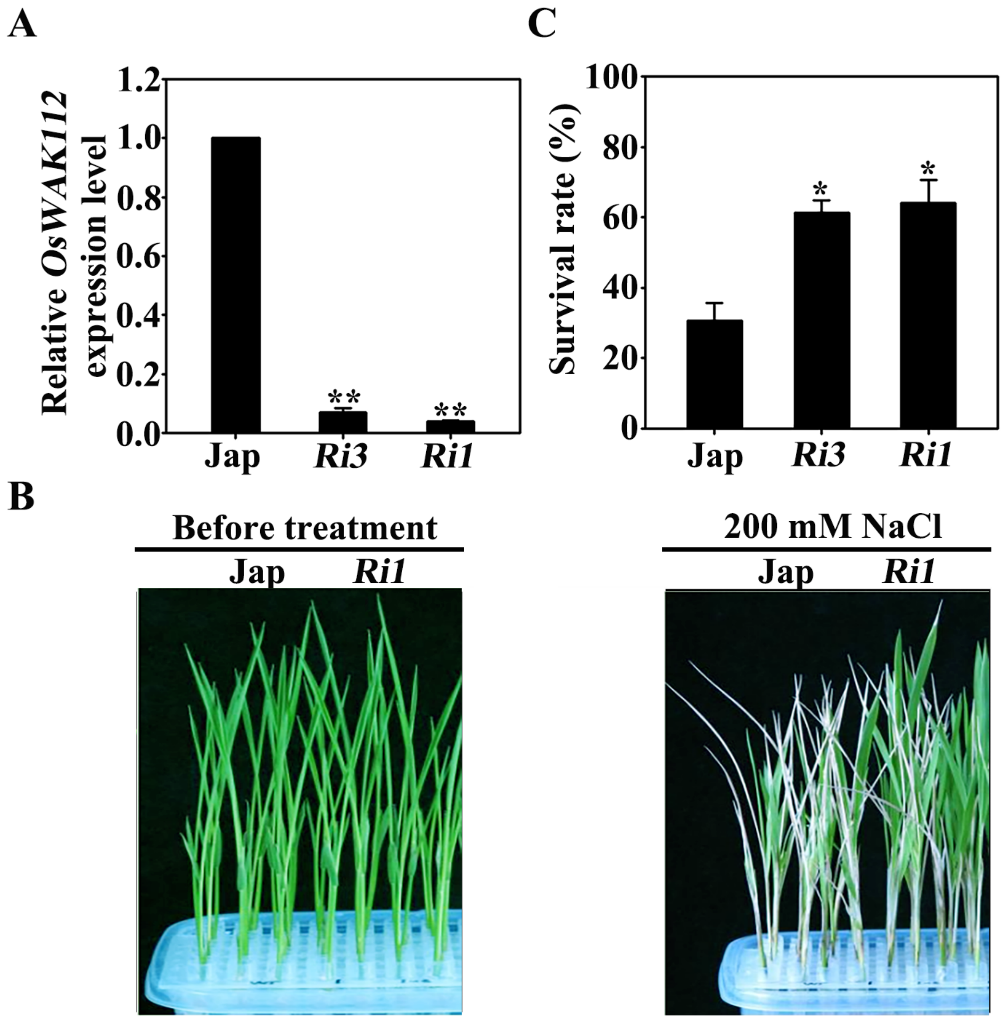

Supplementary Figure S2. Phenotypes of the *OsWAK112 RNAi* lines under salt stress.

(A) The *OsWAK112* transcript levels in 7-day-old Jap and *OsWAK112 RNAi* lines as determined by RT-qPCR. (B) A comparison of the phenotypes of Jap plants and a representative *OsWAK112 RNAi* line (*Ri1*) before and after salt treatment. Ten-day-old seedlings were used as described in the Materials and Methods. (C) Survival rates of Jap and two individual *OsWAK112 RNAi* lines following salt exposure. In (A) and (C) each data point represents the mean ± standard error (SE) from three biological repeats (n ≥ 50). Asterisks indicate a difference relative to Jap (Student's *t*-test, *$P < 0.05$ and ** $P < 0.01$).

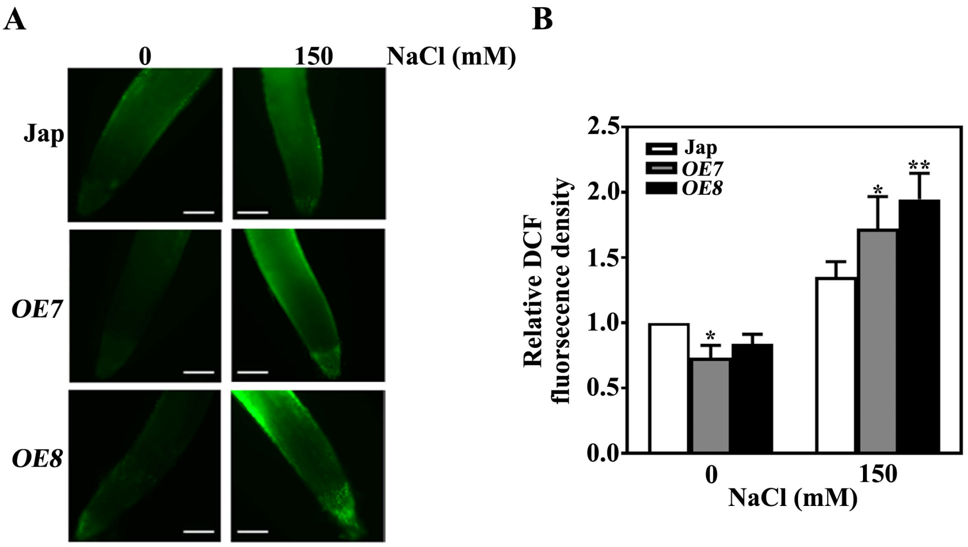

Supplementary Figure S3. ***OsWAK112*** **overexpression affects cellular redox homeostasis in rice.**

(A) and (B) H_2O_2 accumulation in 7-day-old *OsWAK112OE* and Jap seedling roots with or without 30 min of NaCl treatment. Representative images of CM-H_2DCFDA staining (A) showing the ROS level. Scale bar = 100 μM in (A). Quantification of the relative DCF fluorescence intensity (the fluorescence intensity of Jap at 0 mM was set to 1) in the mature zone of the roots is shown in (B). Each data point represents the mean ± SE from three biological repeats (n ≥ 10). Asterisks indicate a difference relative to Jap (Student's *t*-test, *$P < 0.05$ and **$P < 0.01$) in (B).

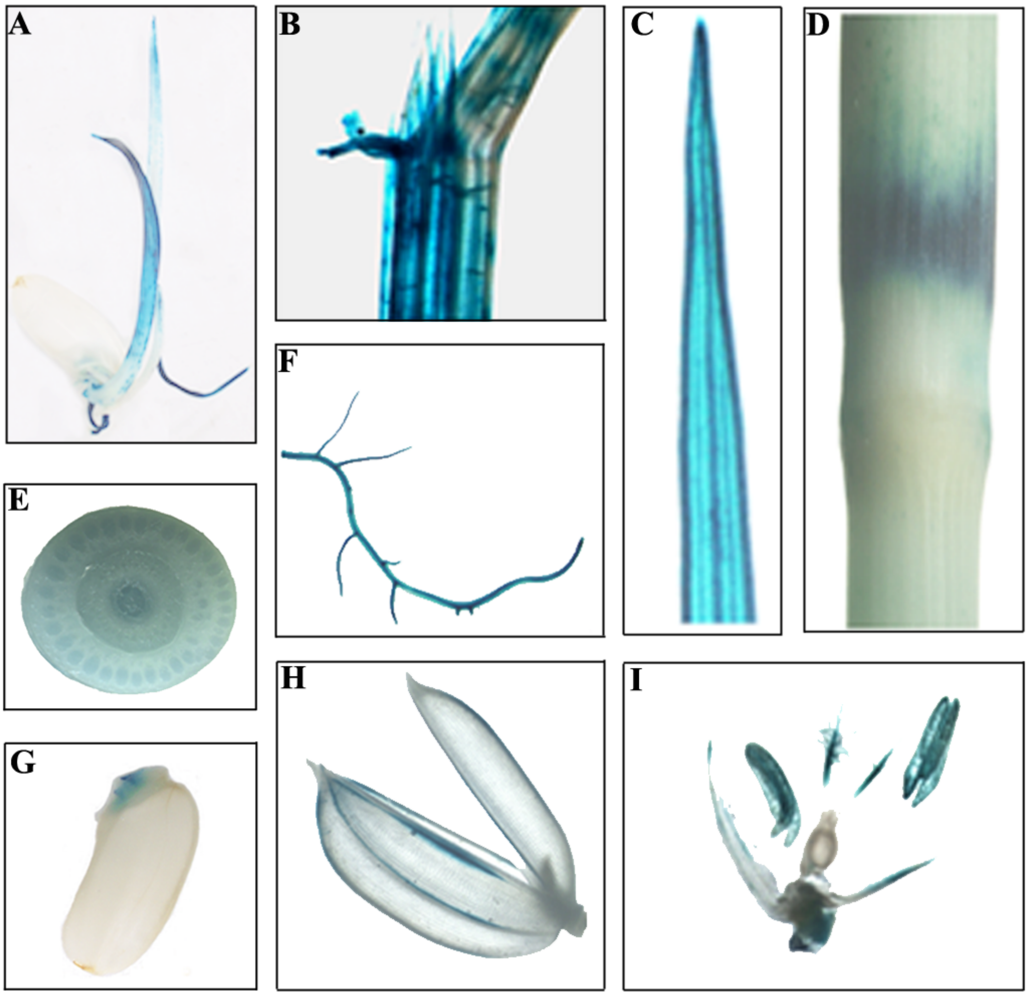

Supplementary Figure S4. OsWAK112 is universally expressed in plants.

OsWAK112 expression in different tissues as detected by GUS staining. GUS staining was observed in coleoptiles (A), ligules (B), leaf blades (C), stems (D and E), roots (F) germinated seeds (G), paleae (H), and stamens and pistils (I).

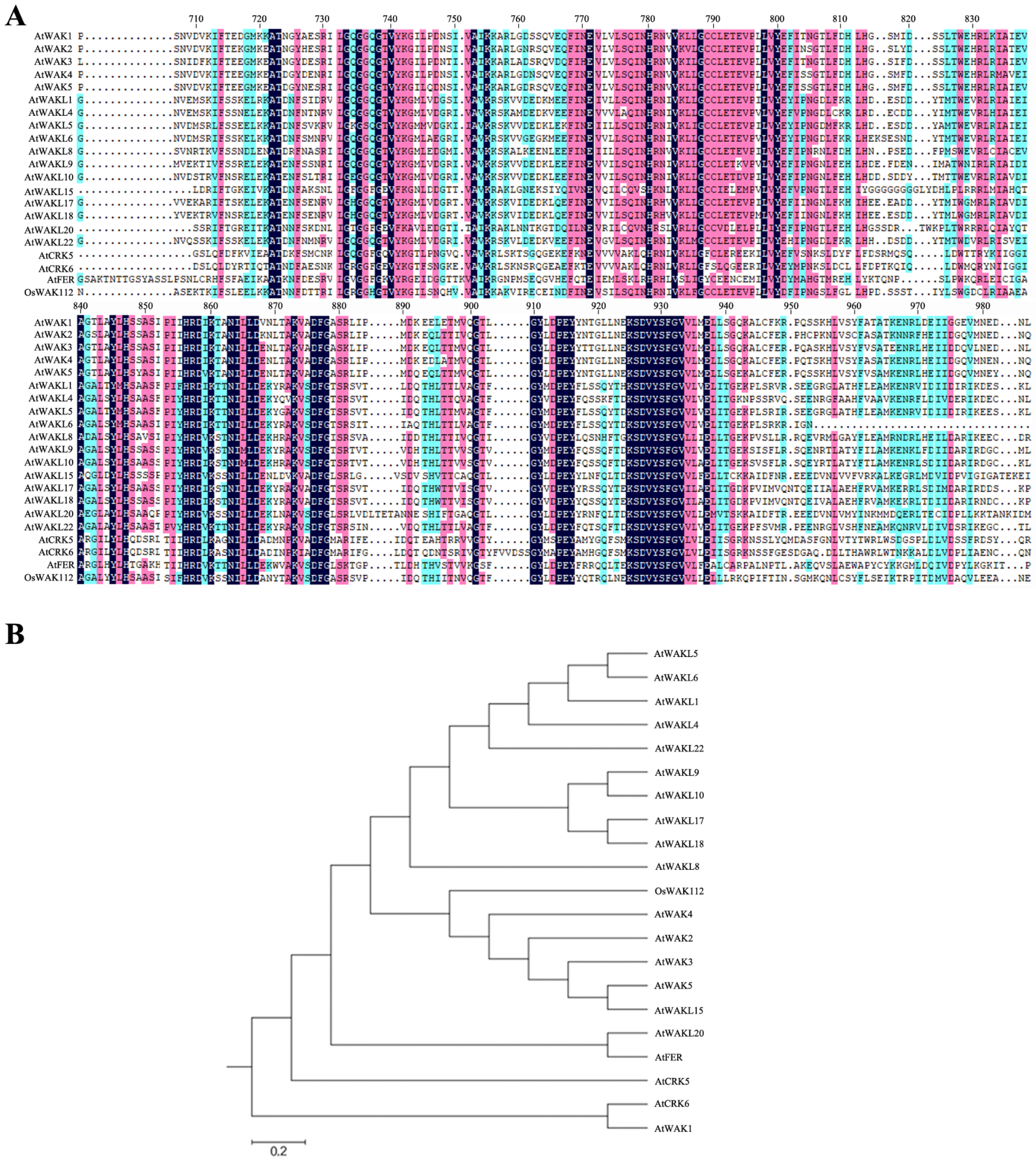

Supplementary Figure S5. Sequence comparison between OsWAK112 and its homologs in *Arabidopsis*.

(A) Similarity analysis of the OsWAK112 kinase domain and its homologs in *Arabidopsis* using a protein sequence alignment. (B) Phylogenetic tree analysis of OsWAK112 and its homologs in *Arabidopsis*. (Both analyses used DNAMAN software.)

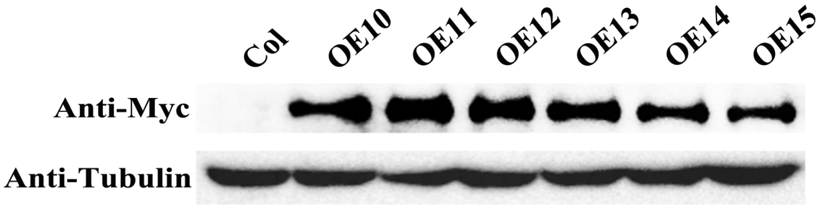

Supplementary Figure S6. Expression of wild-type and mutated forms of OsWAK112 in *Arabidopsis*.

The protein expression levels of OsWAK112 (*OE10* and *OE11*), OsWAK112^{K678E} (*OE12* and *OE13*), and OsWAK112^{D794A} (*OE14* and *OE15*) in 7-day-old *Arabidopsis* plants (Col background) were determined by Western blotting using anti-Myc antibodies.

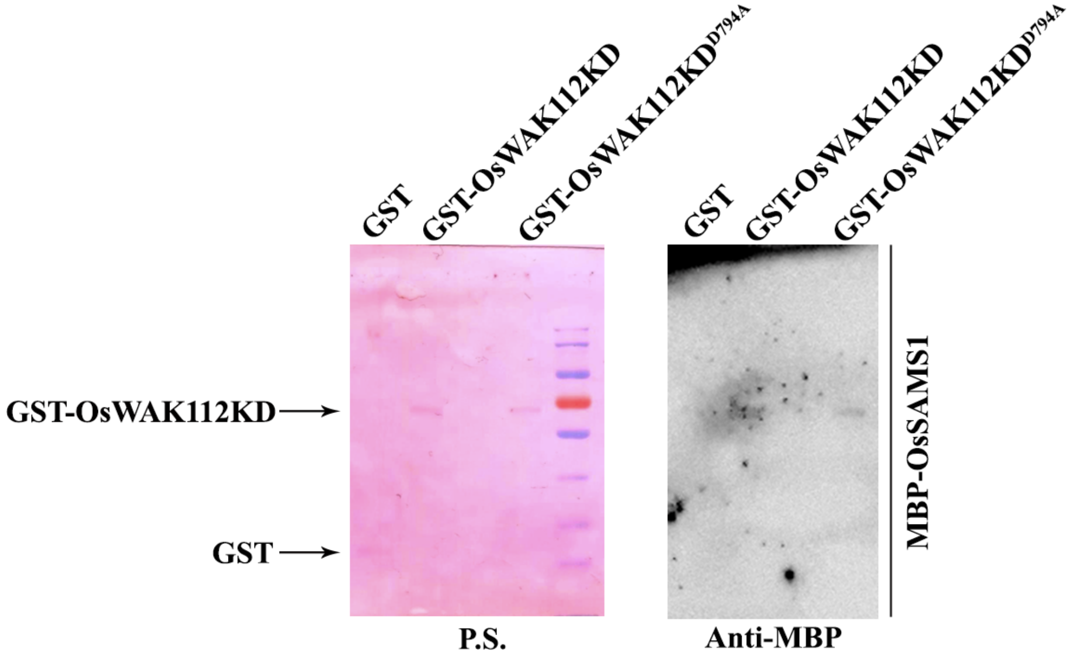

Supplementary Figure S7. GST-WAK112KD^{D794A} interacts with MBP-OsSAMS1 in overlay assay.

MBP-OsSAMS1 bound to the GST-WAK112KD and GST-WAK112KD^{D794A} fusion but not to GST in a gel blot overlay assay. Ponceau S staining indicates equal loading of GST, GST-WAK112KD and GST-WAK112KD^{D794A}. P.S.: Ponceau S.

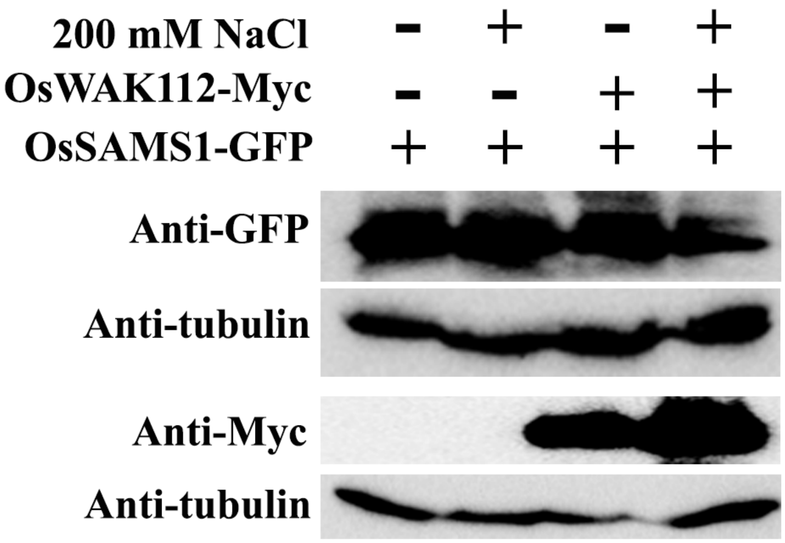

Supplementary Figure S8. OsWAK112 promotes OsSAMS1 degradation under saline conditions in tobacco.

OsSAMS1-GFP alone or co-expressed with OsWAK112-Myc in *N. benthamiana* leaves. Next, the leaves were treated in liquid 1/2 MS medium without (mock) or with 200 mM NaCl for 2 h. Total protein was then harvested for Western blotting. Anti-tubulin was used as an internal loading control.

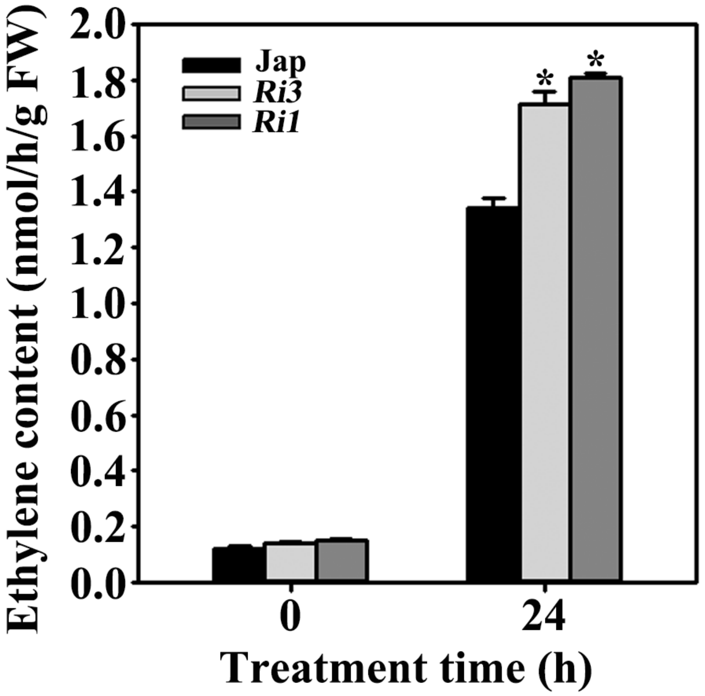

Supplementary Figure S9. OsWAK112 RNAi has high ethylene production under salt stress.

The amount of ethylene per gram fresh weight in 7-day-old Jap, *RNAi1* (*Ri1*), and *RNAi3* (*Ri3*) seedlings grown under normal conditions or treated with 200 mM NaCl for 24 h. Student's *t*-tests were conducted using data from transgenic seedlings compared to those from Jap. Asterisks indicate a significant difference at $P < 0.05$ in each set.

1.2 Supplementary Table 1

Gene number	Primer name	Primer sequence (5'→3')	Purpose
Os10g10130 (OsWAK112)	OsWAK112-F	CACCATGCTTATGTTGTTACTAAACAT	OsWAK112/pENTR recombinant construct
	OsWAK112-R	CCGTGGTAAGCTAGCAGATG	
	OsWAK112-KD-F	TCCCCCGGGGCTTAAAAAGGCGACAAATAAC	GST-OsWAK112KD recombinant construct
	OsWAK112-KD-R	CCGCTCGAGTGGTTGTATCTCTGGGTCACT	
	OsWAK112-K678E-F	TGGCTATTGAAAAGGCCAAAGTTATAAG	GST-OsWAK112KD^{K678E} and OsWAK112^{K678E}/pENTR constructs
	OsWAK112-K678E-R	CTTATAACTTTGGCCTTTTCAATAGCCA	
	OsWAK112-K791R-F	AACTATACTGCTAGAGTTTCAGATTTTGGT	GST-OsWAK112KD^{K791R} construct
	OsWAK112-K791R-R	ACCAAAATCTGAAACTCTAGCAGTATAGTT	
	OsWAK112-D794A-F	TGCTAAAGTTTCAGCTTTTGGTGCTTCA	GST-OsWAK112KD^{D794A} and OsWAK112KD^{D794A}/pENTR constructs
	OsWAK112-D794A-R	TGAAGCACCAAAAGCTGAAACTTTAGCA	
	GUS-F	GCAAGCTTGCCAAATAGCTCTATCACGC	proOsWAK112::GUS construct
	GUS-R	GCGGATCCCCAGACTGAATGCCCACAGGC	
	OsWAK112-QF	CCACTACTCAGCCTCCCAAA	RT-qPCR
	OsWAK112-QR	CCAAACAAGTGGCGCCCC	
	OsWAK112-RNAi-F	GGGGTACCACTAGTGTAGCACTGAGTCAAATCCT	RNAi construct
	Os WAK12-RNAi-R	CGGGATCCGAGCTCTACACCATGTCTGCAATCTC	
Os05g04510 (OsSAMS1)	OsSAMS1-F	CACCATGGCCGCACTTGATAC	OsSAMS1/pENTR recombinant construct
	OsSAMS1-R	TGCAGAAGGCTTCTCCCACTTG	
Os01g22010 (OsSAMS2)	OsSAMS2-F	CACCATGGCGGCGGAGAC	OsSAMS2/pENTR recombinant construct
	OsSAMS2-R	GGAAGATGCCTTCTCATACTTGAGCG	
Os01g18860 (OsSAMS3)	OsSAMS3-F	CACCATGGCTGAGGTTGACACC	OsSAMS3/pENTR recombinant construct
	OsSAMS3-R	TGCAGAAGGCTCCTCCCACTTG	
Os09g00998 (Os18S)	18S–F	TATAGGACTCCGCTGGCACC	RT-qPCR
	18S–R	CCCGGAACCCAAAAACTTTG	
